# The challenge of pain identification, assessment, and management in people with dementia: a qualitative study

**DOI:** 10.3399/bjgpopen20X101040

**Published:** 2020-05-27

**Authors:** Laurna Bullock, Carolyn A Chew-Graham, John Bedson, Bernadette Bartlam, Paul Campbell

**Affiliations:** 1 School of Primary Community and Social Care, Keele University, Keele, UK; 2 Midlands Partnership NHS Foundation Trust, St Georges’ Hospital, Stafford, UK; 3 Family Medicine and Primary Care, Lee Kong Chian School of Medicine, Nanyang Technical University Singapore, Singapore, UK

**Keywords:** analgesics, primary health care, dementia, pain, pain management, community

## Abstract

**Background:**

Painful conditions are common in older adults, including people with dementia. The symptoms associated with dementia (for example, diminished language capacity, memory impairment, and behavioural changes), however, may lead to the suboptimal identification, assessment, and management of pain. Research has yet to qualitatively explore pain management for community-dwelling people with dementia.

**Aim:**

To explore pain identification, assessment, and management for community-dwelling people with dementia.

**Design & setting:**

A qualitative study was undertaken, set in England.

**Method:**

Semi-structured interviews took place with people with dementia, family caregivers, GPs, and old-age psychiatrists. Data were analysed thematically.

**Results:**

Interviews were conducted with eight people with dementia, nine family caregivers, nine GPs, and five old-age psychiatrists. Three themes were identified that related to pain identification and assessment: gathering information to identify pain; the importance of knowing the person; and the use of pain assessment tools. A further three themes were identified that related to pain management: non-drug strategies; concerns related to analgesic medications; and responsibility of the caregiver to manage pain.

**Conclusion:**

Identifying and assessing the pain experienced by people with dementia was challenging. Most people with dementia, family caregivers, and healthcare professionals supported non-drug strategies to manage pain. The minimal concerns associated with non-drug strategies contrasted the multifactorial concerns associated with analgesic treatment for people with dementia. Given the complexity of pain identification, assessment, and management, primary care should work together with family caregivers and community services, with case finding for pain being considered in all assessment and management plans.

## How this fits in

People with dementia are at risk of their pain being inadequately identified and assessed, potentially leading to suboptimal pain management. The majority of people with dementia live in the community, receiving care and support from their family, primary care, and specialist dementia services; however, existing research has yet to explore pain identification, assessment, and management from these perspectives. This study illustrates the complexity in the identification and management of pain for people with dementia, and the unique challenges in community and primary care settings. There is a need for a collaborative approach between the patient and family caregivers, primary care clinicians, specialist dementia services, and other community services (for example, pharmacists), each of which has the opportunity to play a role in pain management in the community.

## Introduction

Dementia is a broad term for a number of conditions with a common set of symptoms that may include memory loss and difficulties with thinking, problem-solving, or language.^1^ Seven per cent of people aged >65 years have dementia,^2^ with the number of people with dementia forecast to rise to >1 million by 2025 in the UK.^2^ Estimates suggest that 50% of people with dementia experience pain, which is in accord with rates in similarly aged people without dementia.^3,4^ Yet pain remains inadequately identified and assessed,^3,4^ and people with dementia receive less pain management than older adults without dementia.^5,6^ While some explanation for this discrepancy may be owing to altered pain experience associated with neurodegeneration, the evidence thus far is limited and inconsistent in this area.^7–10^

While suboptimal pain management can have expected consequences, such as undue distress, discomfort, and activity restriction,^11–14^ there are also additional dementia-specific implications. In particular, pain expression (for example, agitation, aggression) can be misinterpreted as 'behavioural and psychological symptoms of dementia' (BPSD) leading to unnecessary treatment (antipsychotics) of these symptoms, rather than addressing the underlying cause.^15–17^ This is important because antipsychotics are widely used to manage BPSD, despite evidence of limited efficacy and increased risk of adverse events and avoidable mortality.^18^ The National Institute of Health and Care Excellence (NICE) guideline NG97 emphasises the need to consider pain as a potential cause of BPSD.^18,19^

Qualitative research has previously highlighted the complexity of pain identification, assessment, and management for people with advanced dementia at the end of life, or in care home and hospital settings from the perspective of healthcare professionals^20–25^ and family caregivers.^26–28^ However, there is a current lack of research exploring pain for community-dwelling people with dementia,^29–31^ particularly from the perspective of GPs.^29^ This is a significant omission because the majority (>60%) of people with dementia live in community settings.^2^ Therefore, the aim of this study was to explore pain identification, assessment, and management for community-dwelling people with dementia, from the perspectives of people with dementia, family caregivers, GPs, and psychiatrists.

## Method

Semi-structured interviews were used to explore pain identification, assessment, and management in community-dwelling people with dementia. This study was theoretically informed by a critical realist perspective that each individual has their own subjective, real-world lived accounts shaped by culture, history, and experience.^32,33^ Reported findings were guided by the Standards for Reporting Qualitative Research^34^ (see Supplementary Table S1).

### Participants

Research participants were people with dementia, family caregivers, GPs, and psychiatrists. See Table 1 for details of inclusion and exclusion criteria.

**Table 1.** Inclusion and exclusion criteria for people with dementia, family caregivers, and healthcare professionals

**Person with dementia**
Inclusion criteria	
Diagnosis of dementia as assumed by information provided by the agencies involved (that is, Join Dementia Research records), or by verbal confirmation from the person themselves or their caregiver.	
Lives in the community (lives in their own home alone, with family members, in an assisted living facility, retirement community, or residential home).	
Has a named caregiver willing to participate in a dyadic interview.	
Able to vocalise a willingness to take part in an interview.	
Verbally proficient in English.	
Exclusion criteria	
Currently or recently experiencing any major psychological, physical distress, or emotive or stressful life events which taking part in an interview may exacerbate (as asked to the caregiver).	
**Family caregivers**	
Inclusion criteria	
Self-identifies as the current or previous primary caregiver for an individual diagnosed with dementia. This may include a family member, relative, neighbour, or friend who is involved in care or spends the most time with the person with dementia.^35^	
Willing to take part in an interview (alone or with the person with dementia).	
Verbally proficient in English.	
Exclusion criteria	
Aged <16 years.	
**Healthcare professionals**	
Inclusion criteria	
Self-reports as working with people with dementia in a professional capacity.	
Has experience providing care for people with dementia in a professional capacity.	
Willing to take part in an interview.	
Verbally proficient in English.	
Exclusion criteria	
Not directly involved in the provision of care for people with dementia.	

### Setting and recruitment

People with dementia and caregivers were sampled purposively using Join Dementia Research (JDR). JDR is a National Institute of Health Research (NIHR) initiative that allows people to register their interest in taking part in dementia research using an online self-registration service (https://www.joindementiaresearch.nihr.ac.uk).

GPs and psychiatrists were recruited using snowball sampling via existing clinical networks at Keele University.^36^

### Data collection

Interviews were conducted by a single author (LB) between December 2017 and July 2018. Although covering slightly different topic areas for each group, the interview topic guides shared an overall focus around pain identification, assessment, and management (see Supplementary Table S2). Interview topic guides were developed in collaboration with a Patient and Public Involvement and Engagement group of family caregivers of people with dementia and informally piloted with colleagues.

Dyadic semi-structured face-to-face interviews were conducted with people with dementia and their caregiver at their own home. Semi-structured interviews were completed with caregivers alone if the person with dementia was not eligible to participate, or did not wish to participate (see Table 1). Written informed consent was obtained from participants. If the person with dementia did not have capacity to provide informed consent, the caregiver was asked to advise on behalf of the person with dementia as a consultee.^37^ The ‘process consent’ method used in this study ensured a person-centred approach to consent for people with dementia.^38–40^

Sociodemographic characteristics (for example, age, sex, Mini-Mental State Examination [MMSE] score) were obtained from JDR records (where available) and/or discussed at the start of the interview. Additionally, the person with dementia provided a self-report of their pain, and the caregiver provided an informant report of the pain experienced by the person with dementia (current pain and pain during the previous 4 weeks) using the Revised Iowa Pain Thermometer (IPT-R).^41,42^ The IPT-R not only provided important contextual information that was reflected on by LB during the analysis, but also prompted participants to qualitatively reflect on self-report pain tools for people with dementia.

Semi-structured interviews were conducted with GPs and psychiatrists, either face to face or by telephone. If face to face, interviews were completed at the healthcare professional’s practice or at the university, and written informed consent was obtained. If by telephone, written informed consent was completed by the participant and returned to the interviewer prior to interview.

### Analysis

Interviews were digitally recorded, transcribed verbatim, and anonymised. Data collection and analysis were undertaken concurrently so that topic guides could be modified iteratively. Data were analysed inductively using reflexive thematic analysis to explore patterns of shared meaning across the dataset.^43,44^ NVivo (version 11) software was used to facilitate data analysis and management. Codes and themes were discussed as a multidisciplinary team, including social scientists with research backgrounds in psychology and psychiatry, and academic GPs with expertise in pain, mental health, and comorbidity. The diverse array of research backgrounds contributed to the analysis, interpretation, and ultimately the trustworthiness of analysis.^45^ Initial analysis was conducted within each participant group. After establishing initial codes, each participant group dataset were compared and contrasted to integrate the overall findings. Recruitment ceased when no new themes were identified.^46^

### Reflexivity

Reflexive approaches explored how characteristics of those involved in the data collection and analysis influenced the findings. For example, before the first GP interview, the interviewer perceived the GP as the ‘expert’ owing to her student status and lack of clinical background. This preconception was quickly readdressed as many GPs viewed the interviewer as the expert in pain for people with dementia, using phrases such as *‘you probably already know all of this’*. This may have made GPs cautious to express their feelings, thoughts, and experiences if these deviated from the ‘right answer’. Reflexive practices (for example, a reflexive diary) allowed decisions to be audited throughout the research process.

## Results

Nine interviews took place with people with dementia and/or their family caregiver: seven spousal dyads, one father and son dyad, and one individual interview with a family caregiver (see Table 2). People with dementia (two female, six male), had a mean age of 73.5 (range 57–83) years. Family caregivers (five female, four male) had a mean age of 68 (range 52–82) years. Brenda participated with her husband but also reflected on pain management for her mother with dementia who lived alone. All reflected on recent pain experienced by the person with dementia; four had osteoarthritis; two had multiple comorbidities.

**Table 2.** Demographic details of interview participants with dementia and family caregivers of people with dementia.

**Pseudonym**	**Relationship**	**Age, years**	**Previous occupation**	**Diagnosis** (date)^c,d^	**Cognitive test score** (test, date)^c,^ ^d^	**Current pain conditions** ^c,d^	**Pain now^e^**	**Pain in previous 4** weeks^e^
Patricia^a^	Wife	82	OT	AD (2014)	18/30 (MMSE, 2016)	Spinal injury	Moderate	Moderate
Robert	Husband	82	Aviation	Caregiver	–	–	Unable to judge	Unable to judge
James^a^	Husband	74	Purchaser	AD (2016)	87/100 (ACE-R, 2016)	Neck pain	None	None
Mary	Wife	74	PA	Caregiver	–	–	None	None
Barbara^b^	Wife	67	Teacher	PCA	–	Neuralgia, osteoporosis	–	–
John	Husband	67	Electrician	Caregiver	–	–	Severe	Moderate
William^a^	Husband	78	Lecturer	AD (2012)	24/30 (MMSE, 2014)	Osteoarthritis (knees)	Mild	Mild
Carol	Wife	74	Probation officer	Caregiver	–	–	None	Mild
Richard^a^	Father	83	Tile making	Mixed (2015)	Unknown	Osteoarthritis (back)	Moderate	Moderate
David	Son	52	Catering^f^	Caregiver	–	–	None	Mild
Mark^a^	Husband	73	Probation officer	AD (2014)	81/100 (ACE-R, 2014)	Tooth pain	None	None
Brenda	Wife	68	Nurse	Caregiver	–	–	None	Mild
Steven^a^	Husband	57	Postman	Mixed/FTD (2017)	Mild (MMSE, 2017)	Back pain	None	Mild
Michelle	Wife	53	Caregiver	–	–	None	Mild
Linda^a^	Wife	77	Administrative	FTD (2017)	25/30 (MMSE, 2017)	Osteoarthritis, gout	Mild	Unanswered
Charles	Husband	77	Optometrist	Caregiver	–	–	Moderate	Moderate
Greg^a^	Husband	64	RAF	Mixed/FTD (2017)	48/100 (ACE-R)	Frozen shoulder, tennis elbow, spondylitis, osteoarthritis (hips)	Moderate	Moderate
Denise	Wife	66	Shop assistant	Caregiver	–	–	Moderate	Moderate

^a^Person with dementia. ^b^Person with dementia did not participate in the interview. ^c^Information obtained from Join Dementia Research records. ^d^Information obtained during the interview (from person with dementia or caregiver). ^e^The pain experienced by the person with dementia using the Revised Iowa Pain Thermometer. ^f^Currently employed. ACE-R = The Addenbrooke's Cognitive Examination Revised. AD = Alzheimer’s disease. FTD = frontotemporal dementia. MMSE = Mini-Mental State Examination Score. OT = occupational therapist. PA = personal assistant. PCA = posterior cortical atrophy. RAF = Royal Air Force.

Nine GPs (four female, five male) and five psychiatrists (all female) completed an interview. Years of experience working in their current profession ranged from 1 year to 33 years (see Table 3). Interviews lasted from 31 to 120 minutes.

**Table 3.** Details of interview participants: healthcare professionals.

**Pseudonym**	**Sex**	**Current profession**	**Year(s) of experience^a^**
Tom	Male	GP	12
Alan	Male	GP	33
Jenny	Female	GP	30
Jessica	Female	GP	1
Muhammad	Male	GP	3
Lisa	Female	GP	11
Ishann	Male	GP	1
Amy	Female	GP	5
Chris	Male	GP	7
Prisha	Female	Consultant — old-age psychiatry	10
Hayma	Female	Consultant — old-age psychiatry	8
Aska	Female	Associate specialist — old-age psychiatry	11
Rina	Female	Consultant — old-age psychiatry	3
Mel	Female	Associate specialist — old-age psychiatry	18

a In current role.

**Figure 1.**
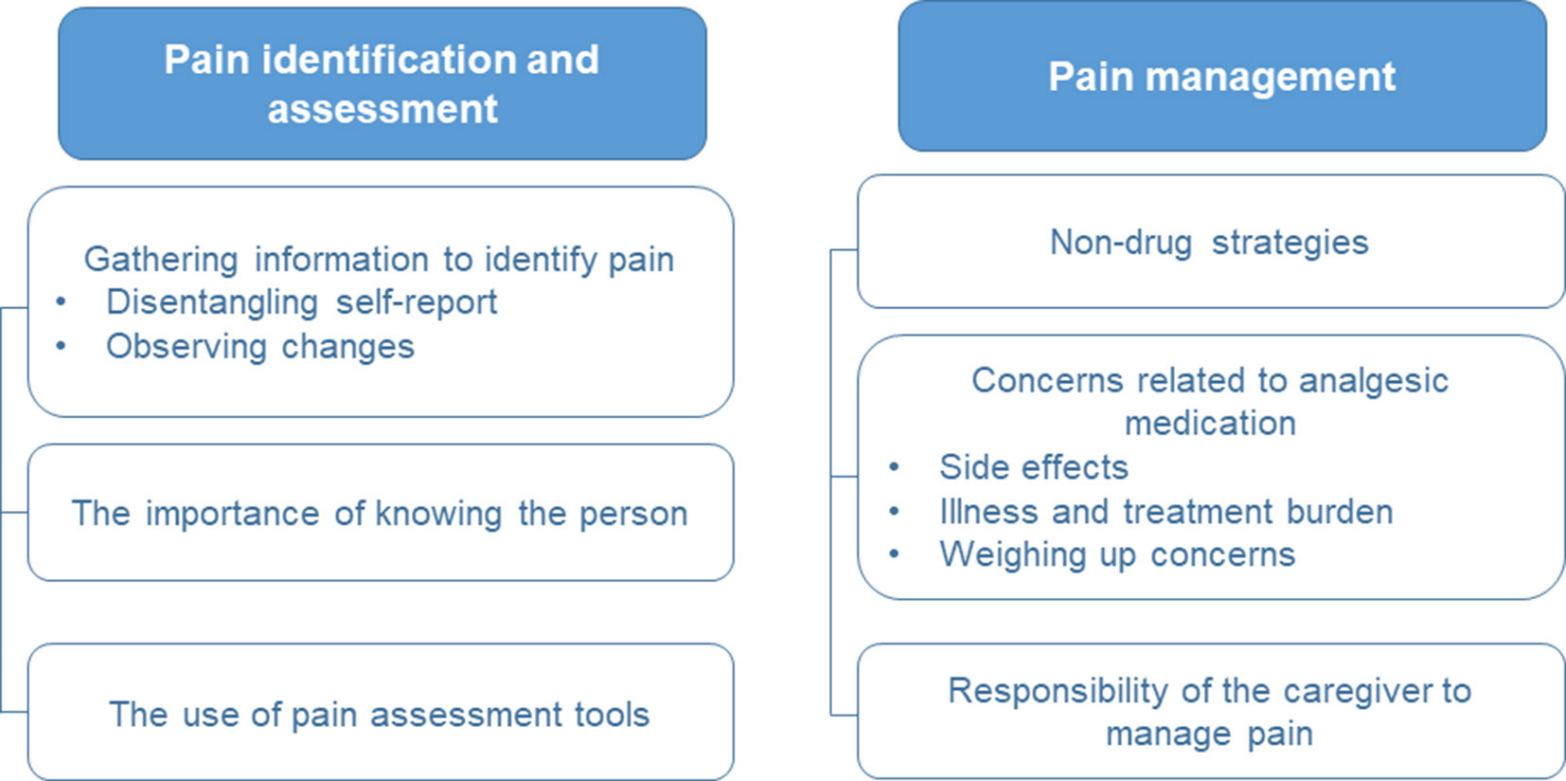
Themes and subthemes identified in the data

Data were organised into six main themes. Three themes related to pain identification and assessment, and three themes related to pain management (see Figure 1).

Details of the themes are presented using illustrative quotations. Unique identifiers for people with dementia (PWD) and their caregivers (CG) include their pseudonym, their sex (male [M] or female [F]), age, and self-reported pain severity. For healthcare professionals, the unique identifier includes their pseudonym, professional group (GP or psychiatrist [P]), and years of experience.

### Gathering information to identify pain

To identify the experience of the people with dementia, healthcare professionals gathered information in an attempt to build a comprehensive picture. However, this process was perceived as a complex task associated with uncertainty:

*'I think it's difficult, because it's trying to untangle a ball of wool* *.* *'* (Alan, GP, 33)

Self-report was an integral yet often challenging method to gather information about pain, especially as dementia progressed:

*'... it's more the severe end that I’m talking about here, where it's difficult having a conversation with someone, and sort of answering those* [self-report and history] *questions can sometimes be a tricky area.'* (Chris, GP, 7)

A number of caregivers, including David (whose father reported moderate pain at the time of the interview), questioned if self-report reflected pain experienced by the person with dementia:

*'Well sometimes you feel it* [discomfort] *in your back, don't you? But it only seems to be a problem when I want you to go and do something.'* (David, CG, M, 52)

Relying on the person with dementia to mention their pain was challenging if there was inconsistency between self-report and caregiver report:

*'Lots of patients that will be brought in by family members and they're saying the patient is really struggling with knee pain, and then you talk to the patient and they're saying "oh no I’m fine, everything is okay" and it's that inconsistent history that makes the assessment challenging.'* (Amy, GP, 5)

Dissonance between the person with dementia’s self-reporting of pain and the GP’s clinical assessment was also expressed:

*'I had a lady come in; she'd snapped two bones in her hand, and it was really, really deformed* […] *I said: "is this painful?" "No." "Is this painful?" "No." "Is anything painful?" "No." I don't believe you can fracture two bones* […] *I don't believe that wasn't painful.* *'* (Alan, GP, 33)

An alternative strategy of gathering information to identify pain was observing behavioural, psychological, and physical expressions and/or changes. Family caregivers and GPs were aware that behavioural changes (for example, facial expressions, changes in mood, and body language) may indicate that ‘something else was going on’. However, determining if that ‘something else’ was pain was challenging:

*'... if I've got that distressed patient with dementia, and I think they might be in pain or they might not ... that I think is— is I find that situation far more challenging.* *'* (Ishann, GP, 1)

The challenge of determining the driver of behavioural and psychological changes may mean that behavioural and psychological changes are misattributed to dementia rather than to pain (or some other driver):

*'... dementia with psycho-behavioural disturbance, BPSD, and it's often just put down to the dementia itself.* *'* (Tom, GP, 12)

Psychiatrists expressed that the identification of physical problems (for example, pain) should be prioritised in the presence of BPSD:

*'... whether that's constipation, UTIs, or pain, or whatever, that's always something that we try to go through first before coming to sort of psychiatric reasons of the behavioural or psychological symptoms.'* (Mel, P, 8)

However, psychiatrists also expressed that pain identification was the GP’s responsibility:

*'I personally think that it shouldn't come to a psychiatric nurse, or a psychiatrist for someone with a physical problem, this should have been looked into by their own doctor.* *'* (Aska, P, 11)

In addition to observing behavioural and psychological changes, caregivers observed physical changes that may be indicative of pain:

*'... you think "right let's have a look" and I saw, well it's all swollen, but you have to use your own common sense ... there's no guide.'* (Carol, CG, F, 74)

GPs also recognised the importance of examination to identify physical changes indicative of pain. However, physical examination did not come without its own challenges:

*'You have to go through a fairly detailed ... examination* […] *the trouble with that, quite bluntly, takes a long time ... we're meant to have 10 minutes.* *'* (Alan, GP, 33)

### The importance of knowing the person

Family caregivers reflected on how their familiarity with the person with dementia aided their ability to identify pain:

*'... it's actually the carers that know. If you've been with somebody for 40 odd years you know that person inside out.'* (Brenda, CG, F, 68)

In contrast, many GPs felt that they did not know their patient with dementia:

*'There is a huge problem in terms of continuity, and I think what, you know, the old GP would have been able to pick up as a change in Mrs Blogs, maybe now won’t get noticed.'* (Lisa, GP, 11)

In the absence of familiarity, GPs used the information provided by the caregiver as a ‘surrogate familiarity’:

*'... often the spouse will say, you know, "I'm really worried, and I think they might be in pain" or I think this, or I think that. They're invaluable and insightful in terms of decision making.'* (Jenny, GP, 30)

### The use of pain assessment tools

A number of GPs used self-report methods, but often questioned the accuracy of the answer provided by the person with dementia:

*'Sometimes I use the 1–10 scale* […] *I wouldn't rely on that too much with a person with dementia, because that might not be accurate.'* (Muhammed, GP, 3)

Most GPs were unaware of, or did not use, tools designed for people with advanced dementia (for example, behavioural observation pain assessment tools):

*'I think if there was a specific pain assessment tool of some description ... Which you're probably going to tell me that there is that I don't know about.'* (Lisa, GP, 11)

Only one GP was aware of behavioural observation pain tools; however, preferred other methods to assess pain:

*'The Abbey Pain Scale, that's one scale ... but in my practice* [we] *tend to really go by the observations of carers.'* (Tom, GP, 12)

### Non-drug management of pain

Most people with dementia and caregivers supported non-drug strategies to manage pain. In particular, some reflected on the benefits of physiotherapy, albeit the benefits were sometimes perceived to be short lived:

*'A lot of the exercise I'm doing already … I'm doing them every morning … Even with the exercise they're* [shoulders are] *slowly getting worse ...* *'* (Greg, PWD, M, 64, moderate)

GPs also supported the use of physiotherapy to manage pain, perceiving physiotherapy to have long-term benefits, especially when compared with the short-term relief of analgesic medication:

*'Any exercises they can do, things they can do at home* […] *in the long term*, *keeping the joint active is much more beneficial than putting someone on co-codamol*.*'* (Amy, GP, 5)

In addition to physiotherapy, non-drug treatments that provided warmth and comfort (for example, wheat bags, hot-water bottles), along with massage and distraction techniques (to divert attention away from the pain) were used by caregivers:

*'... she always has a hot-water bottle at night, so she's always warm and comfortable, and all of that helps ... common sense really.'* (Brenda, CG, F, 68)

Many of these strategies were also supported by GPs:

*'Other things that can be really helpful that I suggest often is massage.* […] *heat packs, cold packs … those are nice and safe aren't they, and easier? Or distraction techniques, you know? Rather than focusing on the pain, trying to go off and do something like listen to the radio.'* (Lisa, GP, 11)

A small number of caregivers reflected on their use, yet there was scepticism of alternative and complementary medicines. John perceived his wife to experience severe pain, potentially illuminating his desperation to try such treatments in spite of his scepticism:

*'I mean we've thrown money at it … An acupuncturist was recommended to us … Well I mean people have mentioned mindfulness* […] *some of it just sounds rather mad new-age stuff to me* […] *you might regard them as quackery.'* (John, CG, M, 67)

GPs did not recommend alternative and complementary medicines owing to concerns regarding accessibility, financial barriers, and the lack of evidence underpinning their use for pain:

*'As doctors we can't prescribe those things, because they're not necessarily accessible on the NHS* […] *The other thing is the cost implications* […] *it’s often deemed a bit unethical to tell patients "you must have this" knowing that it's going to cost them £30 a go.* […] *I think as practitioners you have to practice evidence-based medicine.'* (Jenny, GP, 30)

Although GPs felt unable to recommend such treatments, they often continued to support their use if suggested by their patient:

*'There's a whole host of other complementary medicines … I generally don't offer them ... Generally, I don't suggest them* [...] *but my personal perspective I support it, I say yeah, you can try it.'* (Ishann, GP, 1)

### Concerns related to analgesic medication

Some people with dementia and caregivers voiced no explicit concerns towards analgesic medications, while many others were reluctant to use them:

*'I hate taking tablets at the best of times, so I've got to be getting pretty bad before I'll take them … I've got an aversion to taking poisons ... Every tablet is a poison of some kind.'* (Greg, PWD, M, 64, moderate)

GPs also acknowledged concerns when prescribing analgesic medication, with a preference for simple analgesics rather than non-steroidal anti-inflammatory drugs and opiates:

*'... this group of patients that are particularly susceptible to side effects of certain drugs so you have to be careful, so you go with a drug which is the simplest ... with the lowest side effect profile, and the staple one is paracetamol.* […] *It's a well-recognised fact that these people get into trouble with non-steroidal anti-inflammatories. Okay let’s move away from that, let’s go to ... narcotic based* […] *so then they get constipated* […] *if you look at it, in those terms, you're really, really limited.'* (Alan, GP, 33)

In addition to side effects, another concern was linked to comorbid conditions and their associated treatments, which may mean that certain analgesics are contraindicated:

*'He can only take paracetamol 'cause he's on warfarin; he's very limited as to what other drugs he can take ... He can't take things like ibuprofen ...* *'* (Carol, CG, F, 74)

The number of medications used by the person with dementia may also increase concerns when adding analgesics into their regimen:

*'He’s on 10 tablets a day anyway, for his various conditions, so painkillers are over and above ...* *'* (Denise, CG, F, 66)

The multifactorial concerns associated with analgesic medication (including side effects, illness, and treatment burden) meant that GPs carefully weighed up their treatment options, considering if the advantages (potential pain relief) outweighed the potential disadvantages (for example, side effects):

*'We would talk about the risks and benefits, and there's— it's not an absolute contraindication to give it to them. It could be worth a small trial, but you have to make clear that it does come with some possible side effects.'* (Muhammed, GP, 3)

### Responsibility of the caregiver to manage pain

Many caregivers were responsible to prompt non-drug treatments and analgesic use for the person with dementia:

*'I have to get up and get the paracetamol or the ibuprofen for him because he won’t, I have to make him take them.'* (Michelle, CG, F, 53)

In keeping with this, GPs were aware of the important role of caregivers in the management of pain and the use of analgesics, especially when prescribing strong analgesic medication:

*'... say there's Oramorph* [opioid analgesic medication] *in the house, you've got to make damn sure there's someone there can administer it appropriately, who understands how much, how regularly, what to do if there's a problem, what to do if it's not working.'* (Lisa, GP, 11)

In addition to the management of analgesic medication, GPs also relied on caregivers to monitor and feedback information:

*'... there's nobody to monitor it, and there's nobody to feedback to us, whether the patient is improving, if they're taking their medication, if they're not taking their medication. It's cast to the individual patient, which is a bit unfair, or falls to the relatives.*' (Alan, GP, 33)

## Discussion

### Summary

Although not an inclusion criterion, many participants living with dementia self-reported recent painful conditions, while the spouse of the person unable to take part reported her current pain as severe. Nonetheless, this study highlighted a number of complexities in the processes involved in the identification, assessment, and management of pain for people with dementia. In the community, family caregivers were responsible for the day-to-day identification, assessment, monitoring, and subsequent management of pain. Family caregivers and healthcare professionals reflected on the challenge of identifying when a person with dementia might be experiencing pain in the absence of self-report. Healthcare professionals suggested that self-report assessment tools were of limited value and reflected on their lack of familiarity with the person with dementia; relying on the knowledge and experience of the caregiver. Family caregivers and healthcare professionals described awareness of behavioural, psychological, and physical changes that may be indicative of pain for people with dementia; however, GPs and psychiatrists expressed concern that pain remained inadequately considered as part of the differential diagnosis of BPSD.

Most participants across all groups supported non-drug strategies as a safer approach to pain management in contrast to the multifactorial concerns associated with analgesic treatment.

### Strengths and limitations

This study provides a rich understanding of how key stakeholders perceive pain identification, assessment, and management, and the unique challenges in primary care and community settings. The study sample was diverse with respect to age, type, and stage of dementia, with healthcare professionals having a broad range of experience.

Dyadic interviews, including the person with dementia and their caregivers, facilitated a safe and comfortable environment for open and honest discussion.^47,48^ However, on reflection, in some circumstances the perspective of the person with dementia was sometimes expressed by the caregiver, on behalf of the person with dementia, despite the person with dementia having the ability to express their own perspective. Additionally, none of the people with dementia lived alone; therefore, findings may not be relevant to that group.

### Comparison with existing literature

Findings are consistent with previous work while highlighting the unique challenges in community and primary care settings.

Many caregivers and GPs in this study perceived self-reported pain as a challenge, especially as dementia progressed, with the presence of dementia making pain assessment difficult.^29,49^ Of the eight dyadic reports, there were inconsistencies in three, in which two caregivers reported a lower severity of current pain compared with the self-report of the person with dementia. In line with this finding, many GPs questioned the ‘reliability’ or ‘accuracy’ of self-report pain tools for people with dementia. This finding is reflected in previous qualitative literature, in which nurses perceived pain assessment as a ‘complex process’,^50,51^ with reduced or altered verbal communication as a key barrier to pain assessment,^30^ and the use of self-report tools.^27,52^

Identifying behavioural, psychological, and physical changes has previously been recognised as a key strategy to build a picture of the pain experience.^30,49^ However, in this study, GPs often found it challenging to determine if pain was the driver of behavioural or psychological changes. This challenge may be intensified as many GPs in this study were unaware of and/or did not use behavioural observation pain tools, despite being recommended by UK guidelines.^19,53^ This finding aligns with previous literature that also found healthcare professionals had a limited awareness or did not use behavioural observation pain tools,^27,29,54^ perceiving them to have limited value.^20^ A meta-review examining the psychometric evidence of behavioural observation pain tools suggests that no one tool is more reliable or valid than the others.^55^ This finding is important as BPSD may ‘mask’ the identification of pain; leading to inadequate pain identification and assessment in primary care.^56^

Familiarity or ‘knowing’ the person with dementia has previously been recognised as important in care home and hospital settings,^22,30^ with caregivers acting as ‘messengers on behalf of the patient’.^28^ This study builds on previous literature by highlighting the unique challenge of patient—doctor relationship continuity in primary care, and the responsibility for the family caregiver to manage pain in the community. To exemplify, in care home settings, studies have examined structured, stepwise interventions with regular pain evaluation to manage pain for people with dementia.^57^ However, in the community the responsibility to perform ‘regular pain evaluation’ would often fall to the relatives of the person with dementia, which may be burdensome without adequate support.

Previous qualitative^51,58,59^ and questionnaire studies^29,49,60^ highlight the importance of non-drug strategies; reflecting on their efficacy and appropriateness. The present study complemented these findings, adding additional insight into the hierarchical perspective, with physiotherapy being perceived in a higher regard than complementary approaches.

This study also found multifactorial concerns associated with analgesic medications, again reflecting existing evidence in care home settings, where analgesic medication was reported as ‘complex’ and ‘restricted’.^28,61^ This study found that GPs weighed up their concerns related to each analgesic; trialling analgesic medication to identify if the advantages (potential pain relief) outweighed the potential disadvantages (for example, side effects). In previous research, similar approaches were used, describing this process as a ‘balancing act’.^25,62^ This finding illuminates the challenge of prioritising pain and pain management amid many potential comorbidities that also require medication.^63,64^

This study highlighted the responsibility and potential burden on caregivers to manage pain in community settings. These findings are comparable to previous qualitative work.^65^

### Implications for practice

Early identification and effective interventions can moderate the impact of chronic pain.^66^ When comorbid with dementia, such an approach to pain may have substantial implications for the individual, society, and for care costs. Given the mounting evidence associating dementia with musculoskeletal conditions,^67^ case finding for pain should be considered in any assessment and management plan. Guidelines recommend a multidimensional assessment of pain (including self-report, observation, and informant reports) for people with dementia.^53,68^ Conducting a multidimensional assessment may, however, be challenging within a time-limited GP consultation. Further awareness raising for both caregivers and healthcare professionals is required, highlighting the priority of exploring pain presence taking into account the challenges, especially in the absence of self-report and when BPSD are present. GPs may benefit from support and training to implement behavioural observation pain tools as part of the multidimensional assessment of pain. As part of this, resources and systems may benefit from a review to support GPs and psychiatric services to work more collaboratively when the person with dementia presents with behavioural or psychological changes.

This research identified concerns relating to analgesic medications for people with dementia, including side effects, interactions, and polypharmacy. This highlights the necessity for regular medication review and evaluation of the need for continuing medication, appropriate dose, potential side effects, interactions, and function, in collaboration with caregivers. There is a potential role for pharmacists situated in general practices to work with GPs in the management of medications for people with dementia.^69^ Non-drug interventions or provision may be limited in community settings; however, simple at-home techniques (for example, massage, hot-water bottles, and exercises) should be explored with caregivers. Finally, GPs should remain mindful of how best to identify family-centred needs in order to best support caregivers to identify and manage pain in those in their care.
